# Identification of Transmission Routes of *Campylobacter* and On-Farm Measures to Reduce *Campylobacter* in Chicken

**DOI:** 10.3390/pathogens9050363

**Published:** 2020-05-09

**Authors:** Sara Frosth, Oskar Karlsson-Lindsjö, Adnan Niazi, Lise-Lotte Fernström, Ingrid Hansson

**Affiliations:** 1 Department of Biomedical Sciences and Veterinary Public Health, Swedish University of Agricultural Sciences, P.O. Box 7036, SE-750 07 Uppsala, Sweden; Lise-Lotte.Fernstrom@slu.se (L.-L.F.); Ingrid.Hansson@slu.se (I.H.); 2 SLU Global Bioinformatics Centre, Department of Animal Breeding and Genetics, Swedish University of Agricultural Sciences, P. O. Box 7023, SE-750 07 Uppsala, Sweden; Oskar.E.Karlsson@slu.se (O.K.-L.); Adnan.Niazi@slu.se (A.N.)

**Keywords:** broiler, *Campylobacter coli*, *Campylobacter jejuni*, campylobacteriosis, cgMLST, chicken, environmental sampling, on-farm measures, transmission routes, whole-genome sequencing

## Abstract

An in-depth analysis was performed on Swedish broiler producers that had delivered chickens with *Campylobacter* to slaughter over several years, in order to identify possible transmission routes and formulate effective measures to prevent chickens being colonized with *Campylobacter.* Between 2017 and 2019, 626 samples were collected at farm level and *Campylobacter* was isolated from 133 (21.2%). All *C. jejuni* and *C. coli* isolated from these samples were whole-genome sequenced, together with isolates from the corresponding cecum samples at slaughter (*n* = 256). Core genome multi-locus sequence typing (cgMLST) analysis, using schemes consisting of 1140 and 529 genes for *C. jejuni* and *C. coli*, respectively, revealed that nearby cattle, contaminated drinking water, water ponds, transport crates, and parent flocks were potential reservoirs of *Campylobacter.* A novel feature compared with previous studies is that measures were implemented and tested during the work. These contributed to a nationwide decrease in *Campylobacter-*positive flocks from 15.4% in 2016 to 4.6% in 2019, which is the lowest ever rate in Sweden. To conclude, there are different sources and routes of *Campylobacter* transmission to chickens from different broiler producers, and individual measures must be taken by each producer to prevent *Campylobacter* colonization of chickens.

## 1. Introduction

*Campylobacter* spp. is the most reported bacterial cause of gastrointestinal disease in humans in Sweden, and in other countries [1,2]. In Sweden, a total of 8132 campylobacteriosis cases were reported in 2018, corresponding to an incidence of 80 cases per 100,000 inhabitants, of which 45% were domestically acquired [3]. In the European Union (EU), 246,571 human cases were reported in 2018, corresponding to an incidence of 64 cases per 100,000 inhabitants [1]. However, the number of cases can be assumed to be significantly underreported [4]. For Sweden, a multiplier of 9.05 is suggested, which would mean that the actual number of people affected by campylobacteriosis during 2018 was 73,595 people, at a cost to society of approximately SEK 649 million (SEK 8825 per case) [5]. 

Chicken and chicken products are known to be the major sources of *Campylobacter* infection in humans, through insufficient heat treatment or cross-contamination in the kitchen [1,6]. *Campylobacter* spp. do not multiply in food, but multiplication is not required to cause disease, as the infective dose is very low. In experimental studies, 500–800 CFU of *Campylobacter jejuni* have been shown to be sufficient to cause symptoms in humans [7,8]. The most common *Campylobacter* spp. associated with human infection is *C. jejuni* [1], although cases with other *Campylobacter* spp., for example *C. coli* and *C. lari,* also occur [9]. *Campylobacter* spp. can be present in the intestines of chickens, often in very large quantities (up to log 8 CFU per g feces), without the bird showing any clinical signs of disease [10,11,12]. If live chickens carry the bacteria in their intestines, broiler carcasses and chicken meat may become contaminated with *Campylobacter* spp. during slaughter and processing, and *Campylobacter* can later be transferred to humans by handling contaminated meat or eating undercooked meat.

In Sweden, all chicken flocks have been analyzed at slaughter for presence of *Campylobacter* spp. since the Swedish *Campylobacter* program was first initiated by the Swedish Poultry Meat Association in 1991. The purpose of the monitoring program is to reduce the number of chickens colonized with *Campylobacter,* starting at farm level. There was a decreasing trend in the incidence of *Campylobacter* in Swedish chicken flocks between 2000 and 2013, from 20% of flocks to 9%. This decrease was believed to be mainly due to improved biosecurity routines, new broiler houses, and improved skills among farmers [13]. However, in 2014, the proportion of flocks colonized with *Campylobacter* began to increase again and in 2016 the incidence was 15%. The increased incidence of positive flocks might be due to increased production, owing to higher demand for Swedish chicken at retail level. The increase in production has been achieved by various measures, such as shorter periods between flock rotations and thinning, also called partial depopulation, when a broiler flock is slaughtered at two or more occasions with the purpose of having the maximum allowed density of 36 kg per m^2^ for a longer period. 

Seasonal variations in *Campylobacter*-positive chicken flocks, with the highest occurrence in summer months, have been observed in Sweden and many other countries [10,14,15]. Data obtained in the Swedish *Campylobacter* program also show large variations between producers in the incidence of *Campylobacter*-positive flocks. Some producers often (>30% of flocks) deliver chickens with *Campylobacter*, while others almost never deliver chickens with *Campylobacter* [16]. The broiler producers that often deliver chickens with *Campylobacter* have a major impact at consumer level. In addition, they pose a greater risk of contaminating slaughterhouses and transport crates, and thus of spreading *Campylobacter* to other chicken flocks. For example, carcasses from these chickens are more often contaminated with *Campylobacter,* and in larger quantities. Phylogenetic comparison of *Campylobacter* isolates is necessary in epidemiological studies of *Campylobacter*. There is still a lack of knowledge about transmission routes of *Campylobacter* to broiler flocks and survival of *Campylobacter* in and around broiler houses [17]. Genotyping is helpful in assessing the transmission of risk factors and in obtaining a detailed view of where certain sequence types occur more frequently. 

The aim of this study was to perform an in-depth analysis of Swedish broiler producers delivering *Campylobacter-*positive chickens to slaughter, in order to identify possible transmission routes and devise effective measures in primary production to reduce the risk of chickens being colonized with *Campylobacter* spp. The long-term goal was to reduce the presence of *Campylobacter* spp. in broiler chickens, and thereby also the number of campylobacteriosis cases in humans. 

## 2. Results

### 2.1. Bacteriological Analysis

A total of 626 samples were analyzed, and *Campylobacter* spp. were isolated from 133 (21.2%) of these samples. All isolates were identified to species level using MALDI-TOF MS and *C. jejuni* was found to be the most commonly isolated species, followed by *C. coli*, *C. lari*, and *C. lanienae* (Table 1). 

### 2.2. Whole-Genome Sequencing

The 256 whole-genome sequenced *C. jejuni* (*n* = 235) and *C. coli* (*n* = 21) isolates belonged to 47 different multi-locus sequence type (MLST) profiles, of which 12 were novel profiles (Table 2).

The most common sequence type (ST) found in this study was ST-257, with 58 of the *C. jejuni* isolates from three of the four broiler producers (A, C, and D) belonging to this sequence type (ST). ST-583 and ST-918 were the second most common ST, with 16 *C. jejuni* isolates each. However, ST-918 was only found at one broiler producer (A), whereas ST-583 was found at two broiler producers (A and B). The third most common ST was ST-19, which was found at three broiler producers (A, C, and D). Most of the 12 novel MLST-profiles in this study were found only once, but ST-9714 was found in 10 *C. jejuni* isolates, all from broiler producer B (Table 2).

**Figure 1:**
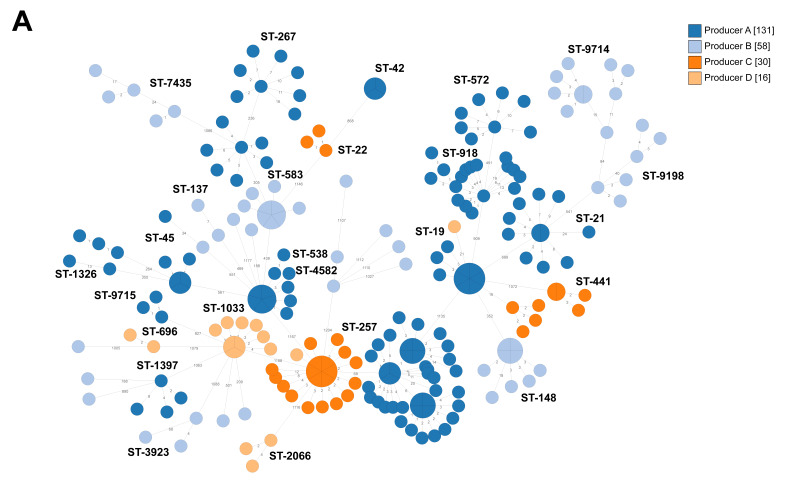

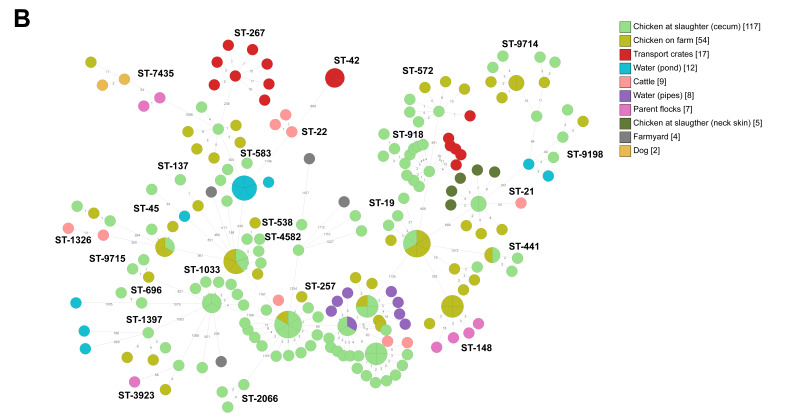
Minimum spanning tree of core genome multi-locus sequence typing (cgMLST) data from *Campylobacter jejuni* isolated from the internal and external environment at four broiler producers in Sweden (A-D) (*n* = 235). Sequence type (ST) is given if at least two isolates share the same ST. The numbers on the lines between isolates represent allelic differences. Line length is not proportional to the numbers. (**A**). Isolates colored according to broiler producer. (**B**). Isolates colored according to sample type.

The core genome MLST (cgMLST) schemes developed for *C. jejuni* and *C. coli* consisted of 1140 and 529 genes, respectively. Sequenced *C. jejuni* isolates of the same ST often clustered together in the cgMLST analysis (Figure 1). However, isolates of ST-45 were diverse, with three different variants being found at broiler producer A and a fourth variant being found at broiler producer B. Isolates of ST-257 that were found at three broiler producers formed two clusters, one for broiler producer A and one for broiler producers C and D (Figure 1). Isolates of ST-583 formed two clusters, one for broiler producer A and B, respectively. Isolates of ST-19 from three broiler producers (A, C, and D) clustered together, but with fewer allelic differences between isolates from the same broiler producer than between different broiler producers. 

#### 2.2.1. Broiler Producer A

**Figure 2:**
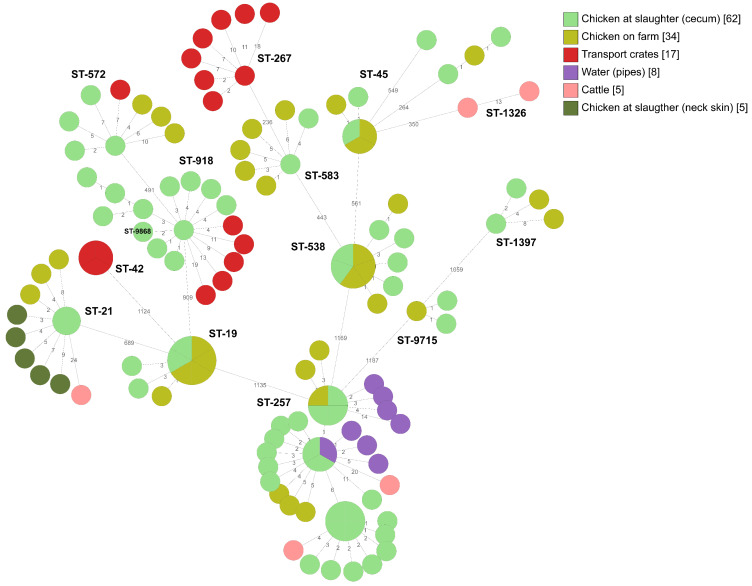
Minimum spanning tree of core genome multi-locus sequence typing (cgMLST) data from *Campylobacter jejuni* isolated from the internal and external environment at broiler producer A (*n* = 131). STs are given for all isolates. The numbers on the lines between isolates represent allelic differences. Line length is not proportional to the numbers.

*Campylobacter jejuni* isolates of several different STs (ST-21, ST-45, ST-257, and ST-572) were found in the internal environment of the broiler houses, in the external environment, and in chickens at slaughter (cecum samples) from broiler producer A (Figure 2). Isolates of ST-257 were obtained from cattle feces samples taken near the broiler houses, from sock samples taken inside the broiler houses, from water from drinking water pipes, and from the cecum samples from 26 slaughter batches during four different flock rotations between January to July 2017. After thorough cleaning of the water pipes in summer 2017, ST-257 was not detected again in any sample from producer A during the study period. In addition to ST-257, ST-21 and ST-1326 were also isolated from feces from the nearby cattle (Figure 2). Furthermore, ST-21 was found in sock samples from inside the broiler house, and in cecum and neck skin samples at slaughter. ST-918 was isolated from cecum samples from more than one flock rotation at slaughter and was also found in transport crates on the arrival at broiler producer A before the chickens were loaded in. Other sequence types isolated from the transport crates were ST-572 and ST-267, of which ST-572 was found in both transport crates and chickens sampled at slaughter (cecum samples). During the 2015–2018 period, *Campylobacter* was isolated from 40% of the slaughter batches delivered by producer A. In the period January–July 2017, *Campylobacter* was isolated from 56% of the slaughter batches. After cleaning the water pipes by increasing and decreasing the pressure of water and air in the water pipes in the end of July 2017, *Campylobacter* was only isolated from 6% of the cecum samples taken at slaughter between August and May 2018. During June to October 2018, the incidence of *Campylobacter*-positive slaughter batches increased to 41%. Since seven different sequence types (ST-19, ST-21, ST-42, ST-45, ST-538, ST-572 and ST-9715) were identified from the chickens during that period it was considered that there had to be more than one reservoir. In addition to regular cleaning between the rotations, changes of socks under the slippers between the departments were introduced in October 2018. This contributed to fact that *Campylobacter* was not isolated, either from the sock samples or the cecum samples, from the 15 slaughter batches in November-December 2018. Furthermore, the incidence was significantly decreased (*p* < 0.00001) to 14% of slaughter batches from producer A in 2019.

#### 2.2.2. Broiler Producer B

**Figure 3:**
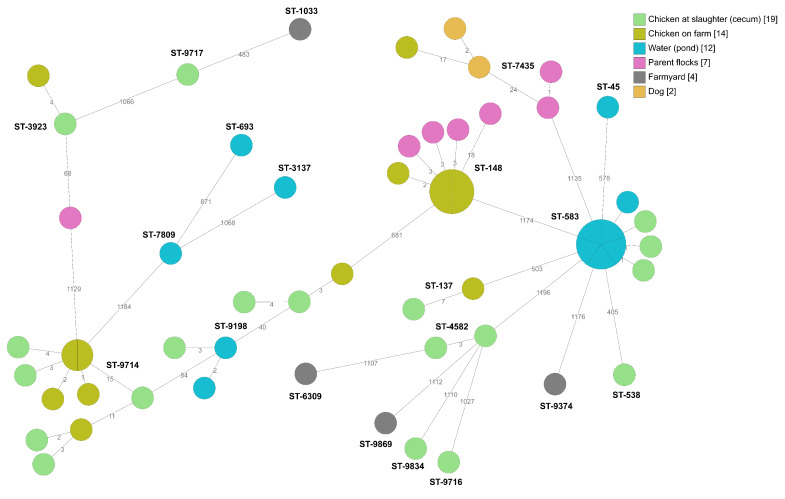
Minimum spanning tree of core genome multi-locus sequence typing (cgMLST) data from *Campylobacter jejuni* isolated from the internal and external environment at broiler producer B (*n* = 58). STs are given for all isolates. The numbers on the lines between isolates represent allelic differences. Line length is not proportional to the numbers.

The result of whole-genome sequencing for *Campylobacter* isolates from producer B reveals a remarkable number (*n* = 30) of different sequence types of *Campylobacter* at farm level and at slaughter (Figure 3). Two *C. jejuni* sequence types, ST-9198 and ST-9714, were detected in chickens on-farm and at slaughter (cecum samples). Three sequence types, ST-148, ST-583, and ST-9198, indicated possible transmission routes for producer B that were not identified at the other producers. ST-148 was isolated from four different parent flocks and from chickens sampled on-farm in all four compartments at one sampling occasion at producer B. In the cgMLST analysis, there were only three allelic differences between three of the isolates from the parent flocks and isolates from the chickens sampled on-farm (Figure 3). *Campylobacter* spp. were isolated from four out of six samples from a water pond next to the farmyard frequently visited by wild birds and wild boar. Whole-genome sequencing resulted in eight different sequence types from the pond, two of which, ST-583 and ST-9198, were also isolated from the chickens (Figure 3). 

**Figure 4:**
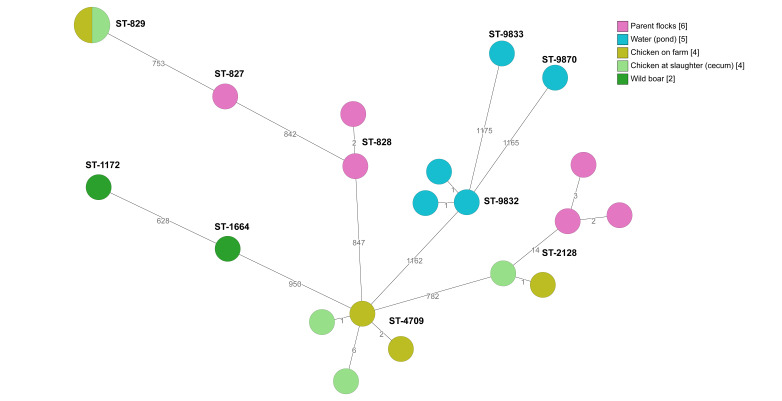
Minimum spanning tree of core genome multi-locus sequence typing (cgMLST) data from *Campylobacter coli* isolated from the internal and external environment at broiler producer B (*n* = 21). STs are given for all isolates. The numbers on the lines between isolates represent allelic differences. Line length is not proportional to the numbers.

Broiler producer B was the only production unit in which *C. coli* was detected. The same *C. coli* isolates, ST-829 and ST-4709, were detected in the chickens on-farm and at slaughter (cecum samples) (Figure 4). In addition, there were only 14 allelic differences between *C. coli* isolates from chicken on-farm, chickens at slaughter (cecum samples), and parent flocks (ST-2178). From 2015 to 2018, *Campylobacter* was isolated from 42% of the slaughter batches delivered by producer B. In 2017 *Campylobacter* was isolated from 57% of the slaughter batches. After installation of protection against wild birds and their droppings above the vent from the broiler house and re-paving the farmyard in December 2017, the incidence was decreased to 46% in 2018. During 2018, the biosecurity was increased in different stages at farm level, such as extending the number of hygiene barriers, including changing footwear three times instead of two, using cotton gloves which were washed after each use. The water pipes were cleaned by increasing and decreasing the pressure of water and air in the water pipes and UV-light treatment of incoming water was installed. Measures were also taken inside the broiler houses such as the sealing of cracks in the floors with silicone and reconstruction of the ventilation on the roof to prevent transmission of condensed water and rainwater to the chickens. In addition, small hatches were installed in the front doors for the removal of dead chickens. The incidence of *Campylobacter* was decreased (*p* = 0.1) to 25% of slaughter batches delivered by producer B in 2019. 

#### 2.2.3. Broiler Producer C

**Figure 5:**
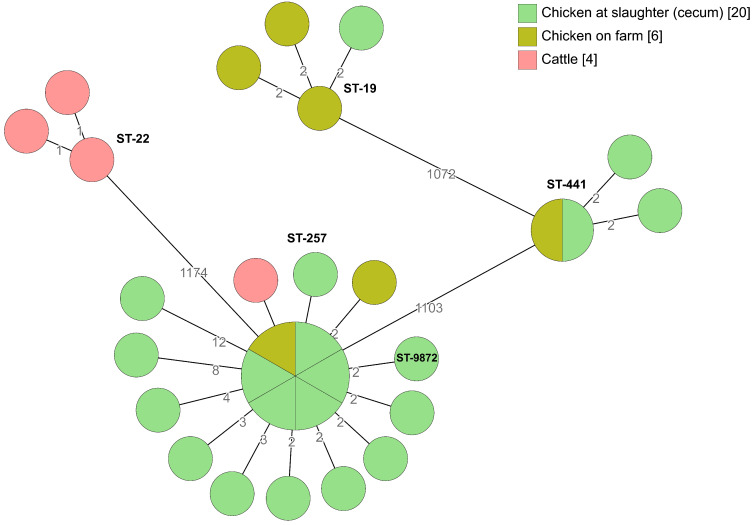
Minimum spanning tree of core genome multi-locus sequence typing (cgMLST) data from *Campylobacter jejuni* isolated from the internal and external environment at broiler producer C (*n* = 30). STs are given for all isolates. The numbers on the lines between isolates represent allelic differences. Line length is not proportional to the numbers.

In the past, broiler producer C has delivered fewer chickens colonized with *Campylobacter* to slaughter than producers A and B. However, during spring–summer 2018, *Campylobacter* was isolated from a greater proportion of slaughter batches from broiler producer C. ST-257 dominated among the sequenced *C. jejuni* isolates and cgMLST analysis revealed that isolates from chickens on-farm, chickens at slaughter (cecum samples), and cattle nearby clustered closely together (Figure 5). This ST-257 was found in 20 of 24 (83%) slaughter batches between May and August from broiler producer C. Different transmission routes were assessed during the visit in August 2018. One of the suspected reservoirs was the nearby cattle. After a fly screen was installed on the roof of the broiler house, the incidence of *Campylobacter* decreased to 14% (eight of 56) slaughter batches delivered by producer C in 2019. 

#### 2.2.4. Broiler Producer D

**Figure 6:**
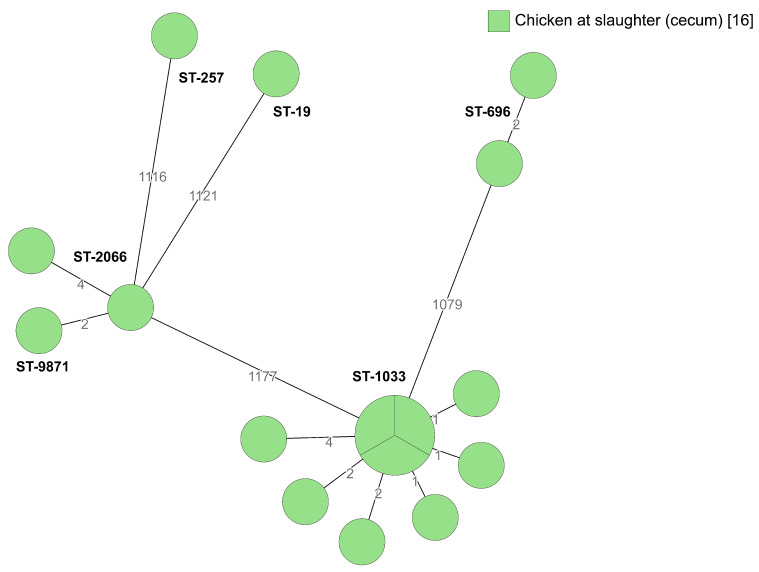
Minimum spanning tree of core genome multilocus sequence typing (cgMLST) data from *Campylobacter jejuni* isolated from broilers belonging to producer D (*n* = 16). STs are given for all isolates. The numbers on the lines between isolates represent allelic differences. Line length is not proportional to the numbers.

Broiler producer D delivers chickens to the same slaughterhouse as producers A and C, but in the past producer D has delivered fewer chickens colonized with *Campylobacter* to slaughter, compared with producers A and B. No environmental samples were collected from broiler producer D, but several different STs (ST-19, ST-257, ST-696, ST-1033 and ST-2066) were isolated from the cecum samples at slaughter, of which ST-1033 was found in different compartments in two consecutive flock rotations on-farm (Figure 6). ST-257 was isolated from cecum samples from one of the flocks, and that isolate differed by only one allele from isolates from producer C (Figure 1). During 2015-2018, *Campylobacter* was isolated from 13% of the slaughter batches delivered by producer D. In the autumn 2018, possible transmission routes such as biofilms in the water pipes and transport crates were assessed and the importance of hygiene barriers such as changing of footwear at least twice and washing hands before working with chicken was discussed. In 2019, the proportion of *Campylobacter*-positive slaughter batches decreased (*p* < 0.00001) to 2%.

## 3. Discussion

The results obtained in this study indicate that the greatest challenge in preventing colonization of broiler chickens by *Campylobacter* is that there are different reservoirs and transmission routes for chicken colonization within and between different producers. Several different reservoirs contributing to *Campylobacter* colonization of chickens were identified in this study. Cattle in a pasture near the broiler houses were a potential reservoir of *Campylobacter* spp. for broiler producers A and C. This is consistent with previous findings that proximity of livestock is a risk factor for chickens being colonized with *Campylobacter* [15,18]. Cattle have also been identified as important reservoirs for human campylobacteriosis, e.g., in the Netherlands, cattle have been identified as the reservoir for 21% of human cases [19], while in Denmark cattle are considered the second most important reservoir [20]. Furthermore, based on MLST data, it has been estimated that 22–55% of all cases of campylobacteriosis in France are caused by ruminants [21]. Since *Campylobacter* are shed in feces and ubiquitous in the environment, including surface waters, they could be transmitted into broiler houses via vectors such as flies, insects, and rodents, or via vehicles as aerosols or dust [22]. It is believed that flies can act as mechanical vectors and transmit *Campylobacter* to broiler houses, and therefore fly screens have been effective in reducing transmission [23]. Fly screens were installed on the roof of the broiler houses at producer C after the same sequence type, ST-257, was identified both in cattle feces and in the chickens, after which the incidence of *Campylobacter*-positive chickens delivered by producer C decreased.

Insufficiently cleaned transport crates is another risk factor for chickens becoming colonized with *Campylobacter* especially during thinning, when birds in a flock are sent to slaughter in separate batches on two or more slaughter occasions [18,24,25]. In this study, 30% of transport crates dispatched by the slaughterhouse were contaminated with *Campylobacter* already on arrival at farm level before loading up the chickens to send to slaughter. The *Campylobacter* isolates from the crates were of several different STs (ST-42, ST-267, ST-572, and ST-918), of which two (ST-572 and ST-918) were also isolated from chickens at slaughter. When transport crates are taken into the broiler houses during thinning, there is a risk of introducing *Campylobacter* to the chickens and continued colonization of the flock for another 5–7 days. Producers A, C, and D deliver chickens to a slaughterhouse that applies thinning. Insufficiently cleaned transport crates could be a possible explanation for the same *Campylobacter* strains circulating among these broiler producers. 

Previous studies have found little evidence of vertical transmission of *Campylobacter* from the parent flocks to the chicken via the eggs [26]. Nevertheless, isolates of the same ST (ST-148) from the parent flocks and the chickens on-farm at broiler producer B clustered closely together in the cgMLST analysis. This particular ST has also been detected in chickens from other broiler producers that received chickens from the same hatchery and in human cases of campylobacteriosis during the same period [27]. Several of the human cases were in people working at the slaughterhouse where chickens originating from those parent flocks were slaughtered [27]. While no evidence has been found to support egg-borne transmission of *Campylobacter* from the parent flock [26,28], it has been suggested that feces containing *Campylobacter* might contaminate the eggshell and shell membranes of freshly laid fertile eggs. With a short period between laying and hatching, together with optimal temperature and presence of moisture when the chicken emerges from the egg, the chicken might ingest feces containing *Campylobacter* and become colonized [29]. Otherwise, the majority of flock colonization results from horizontal transmission from the environment and *Campylobacter* can be easily spread from birds, or other animals, to chickens through a number of routes.

Another potential transmission route detected at broiler producer B was a water pond next to the farmyard that was frequently visited by wild animals. Although several of the *C. coli* STs from the pond and wild boar did not match any of the chicken isolates at the time, *C. jejuni* isolates ST-583 and ST-9198 from the pond matched. Water contaminated with *Campylobacter* is an important source of *Campylobacter* colonization in both broilers and humans [30,31]. *Campylobacter* can survive in water for up to several months, depending on the environmental conditions and on the strain [32,33].

Several of the STs detected in this study have also been isolated from humans and chicken products at retail level in Sweden. For example, ST-19, ST-45, and ST-918 were all detected in humans and in chicken products during the early part of 2017 [34]. ST-918 was also the sequence type responsible for the largest outbreak of *Campylobacter* to date in Sweden (2016–2017) [35]. The most common ST found in the present study was ST-257, which was also the most common ST in humans during August 2018 [36]. ST-257 was isolated from chicken products at retail level during the same period [36], and this ST was found in many reported cases of campylobacteriosis in Sweden during winter 2015 [34]. Furthermore, ST-257 was the second largest cluster in a nationwide Danish study examining around 10% of human cases from 2015 to 2017, where 47% of the clinical isolates formed 104 clusters [37]. In the present study, ST-21, ST-22, ST-42, ST-137, ST-441, ST-538, ST-696, ST-2066, and ST-9198 were detected in chicken and environmental samples and were also found in humans in Sweden during the same period [36,38].

A feature in common to all four broiler producers was that the same *Campylobacter* isolates were detected in several consecutive flocks, i.e., that the *Campylobacter* isolates either survived in the chicken environment or originated from a common reservoir outside the broiler houses. For broiler producer A, the same *Campylobacter* isolate (ST-257) was isolated from the water pipes and from the chickens, indicating that the bacterium had managed to survive in the biofilm inside the water pipes between consecutive flocks. The difficulty in efficiently cleaning water pipes has been mentioned in other studies, which isolated *Campylobacter* from water pipes after disinfection [39,40]. Thorough cleaning of the water pipes by alternating air and water flushing was a measure taken at several of the broiler producers in this study to reduce the risk of next chicken flock being colonized with *Campylobacter* spp. After proper cleaning of the water pipes at broiler producer A, the ST found in water, ST-257, was not found in any of the later chicken flocks. The study also showed that single STs could occur in one flock rotation, but were removed during cleaning and disinfection between flocks. The novel feature of this study compared with previous studies is that preventive measures at farm level were introduced at each producer continuously during the project. In addition, similar measures were introduced at other chicken producers. The results and the measures implemented to improve hygiene barriers were discussed at meetings of broiler producers belonging to the Swedish Poultry Meat Association. As a result, cleaning water pipes by flushing with air and water was performed by producers throughout Sweden that delivered chickens with *Campylobacter* to slaughter in more than two consecutive flock rotations. Since a limited number of broiler producers contributed to the majority of the positive slaughter batches in Sweden [13,16], actions within a few broiler producers have a great impact on the prevalence of *Campylobacter*-positive broilers in the Swedish *Campylobacter* program. Since the actions applied on the broiler producers in this study were also implemented in other broiler producers, the measures contributed to decreasing the proportion of *Campylobacter*-positive flocks at national level from 15.4% in 2016 to 4.6% in 2019 [41], which is the lowest ever rate since the Swedish *Campylobacter* program was started in 1991. At the same time, the rate of reported human campylobacteriosis cases per 100,000 inhabitants in Sweden decreased from 110 in 2016 to 65 in 2019 [42].

The conclusion from this study is that there are different sources and transmission routes for the colonization of chickens with *Campylobacter* at different broiler producers, and thus individual measures have to be taken at each producer to prevent *Campylobacter* colonization.

## 4. Materials and Methods 

### 4.1. Broiler Producers 

Four broiler producers (A–D) were included in the study, each delivering between 70,000 and 480,000 broilers to slaughter around eight times per year. All broilers in the study were conventional broilers without any access to outdoors, and slaughtered at the age of 27 to 37 days. In Sweden, there are about 110 conventional broiler producers in total. Producer A had eight compartments of broilers and *Campylobacter* spp. were detected in 168 of 425 (40%) of producer A’s slaughter batches from 2015 to 2018, in testing within the Swedish *Campylobacter* program. Producer B had four compartments and had *Campylobacter* spp. detected in 52 of 124 (42%) slaughter batches during the 2015–2018 period. Producer C had four compartments of broilers and during the 2015–2018 period, *Campylobacter* was isolated from 36 of 219 (16%) of slaughter batches, with a peak during summer. Producer D had 14 compartments of broilers and between 2015 and 2018, *Campylobacter* spp. were detected in 108 of 844 (13%) of slaughter batches.

Each producer was visited at least once by veterinarians with experience of risk factor analysis of *Campylobacter* in broilers. During the visits, the broiler houses were carefully inspected, including the design of hygiene barriers and the surrounding environment. Possible risk factors and preventive measures to reduce the proportion of chickens with *Campylobacter* were discussed with the producer on these visits. 

### 4.2. Sampling

A total of 626 samples from the internal and external environment of broiler houses, including different water sources, surrounding animals, transport crates, and parent flocks, were collected between 2017 and 2019. Internal and external surfaces were sampled by sock and swab samples, while water was sampled by filtering at least 50 L through a dialysis filter, as previously described [40]. Before taking samples from drinking water pipes, flushing with water and air at increasing and decreasing pressure was performed in all water pipes in the broiler houses. This removed the biofilm inside the pipes, which were included in the samples. The samples from surrounding animals, including wild animals, consisted of either feces or intestinal contents. Samples from fallow deer were collected in connection with hunting and wild boar either in connection with hunting or from fresh feces on the ground. The mice were trapped by mouse traps as part of the broiler producers’ regular pest control. All samples from cattle, dogs, and wild birds consisted of feces samples. Transport crates were sampled at farm level before the broilers were loaded in, and after cleaning and disinfection at the slaughterhouse. 

The sock samples consisted of one pair of sterile cotton tubular retention bandages (>7.5 cm × 25 cm Danafast; Mediplast AB, Malmö, Sweden) moistened with 30 mL Cary-Blair transport medium (SVA321645; National Veterinary Institute, Uppsala, Sweden) and placed over covered boots. The farmer was asked to walk around in the area, making sure all parts of the socks were in contact with feces by turning the socks around the shoe covers. Swab samples consisted of sterile non-woven swabs measuring 10 cm × 10 cm (Shaoxing Yibon Medical Co., Ltd, Shaoxing, Zhejiang, China), which were moistened with 30 mL Cary-Blair transport medium before swabbing of surfaces. After sampling, sock and swab samples were placed in sterile blender bags (Standard 400; Grade Products Ltd, Leicestershire, UK) and a further 30 mL of Cary-Blair transport medium was added to the bag, to keep the samples moistened during transport. Sampling at the farm level was performed both by veterinarians during visits and by the broiler producers. A total number of 343 internal and external samples were collected from broiler producer A at 41 different sampling occasions. Sock samples were collected one to four times from all flocks produced between July 2017 and September 2019. From broiler producer B, 242 samples were collected at 42 different sampling occasions. Sock samples were collected one to three times from all flocks produced between July 2017 and October 2019. From broiler producer C, 41 samples from taken from the environment at six sampling occasions between August and October 2018. Sampling of the parent flocks was performed by sock samples from different parent flocks at five sampling occasions from December 2018 to June 2019. The sampling of parent flocks was targeted to the hatchery delivering chickens to producer B. The samples taken at farm level and parent flocks were transported to the laboratory at the Swedish University of Agricultural Sciences by regular mail, at ambient temperature, and analyses started on the day that the samples arrived at the laboratory.

In Sweden, all flocks sent to slaughter are analyzed regarding the presence of *Campylobacter* within the Swedish *Campylobacter* program [16]. Ten intact cecae from 10 broilers in a slaughter batch were collected after scalding and defeathering, but before washing and cooling of carcasses. The cecae were placed in plastic jars without transport medium and sent by regular mail, at ambient temperature, to the National Veterinary Institute, Uppsala, where they were analyzed as one pooled sample.

### 4.3. Bacteriological Analysis

All samples were cultured for *Campylobacter* spp. according to ISO10272-1 (2017). In brief, all sock and swab samples were enriched in 90 mL (enough to cover the socks) Bolton Broth (CM0983; Oxoid, Basingstoke, UK) supplemented with Bolton Broth Selective Supplement (SR0208E; Oxoid), and incubated at 37.0 ± 1 °C for 4–6 h in a microaerobic atmosphere, followed by incubation at 41.5 ± 0.5 °C for 44 ± 4 h. Two loops of enriched culture (approximately 20 µL) were cultured on modified charcoal-cefoperazone-deoxycholate agar (mCCDA) plates (CM0739; Oxoid), which were incubated at 41.5 ± 0.5 °C for 48 ± 4 h in a microaerobic atmosphere. 

In addition, intestinal contents, feces samples from surrounding animals, and cecae sampled at slaughter were directly cultured on mCCDA plates by taking a loopful of sample. The mCCDA plates were incubated at 41.5 ± 0.5 °C for 48 ± 4 h. The microaerobic atmosphere was generated by CampyGen^TM^ (CN0025; Oxoid) except for the cecum samples, where the microaerobic atmosphere was generated by the Anoxomat system (Mart BV, Lichtenvoorde, Netherlands). Suspected *Campylobacter* colonies were confirmed and identified to species level by matrix-assisted laser desorption/ionization time-of-flight mass spectrometry (MALDI-TOF MS) (Bruker Daltonics, Billerica, MA, USA). In general, one *Campylobacter* isolate was cultured per sample except for the water samples, where several isolates were cultured per sample. All *Campylobacter* isolates identified were stored in Brain Heart Infusion (BHI) broth (CM1135; Oxoid) with 15% glycerol at −70 °C.

### 4.4. Whole-Genome Sequencing

Whole-genome sequencing (WGS) was performed on 256 *Campylobacter* isolates in total, of which 135 were isolated in this study and 121 were isolated previously at the National Veterinary Institute within the Swedish *Campylobacter* program. The isolates from the Swedish *Campylobacter* program were from the same four broiler producers as in this study and from the same period (isolation year in brackets); 62 were from broiler producer A (2017–2018), 23 from B (2017–2018), 20 from C (2018), and 16 from D (2018). A majority of the isolates were *C. jejuni* (*n* = 235), while 21 were *C. coli*.

Genomic DNA was extracted from isolates subcultured twice from single colonies on horse blood agar plates (SVAB341180; National Veterinary Institute) for 48 h at 41.5 °C in a microaerobic atmosphere, using the EZ1 DNA Tissue Kit and the bacterial protocol on an EZ1 Advanced XL (Qiagen, Hilden Germany). The elution volume used was 100 µL and the DNA concentration was measured using the Qubit ds DNA Broad Range assay kit on a Qubit^®^ 2.0 Fluorometer (Invitrogen, Carlsbad, CA, USA). Sample libraries were prepared using the Nextera XT DNA Library Preparation Kit (Illumina, San Diego, CA, USA). Whole-genome sequencing was performed on an Illumina NextSeq 500 system with 2 × 150 bp paired-end reads using the NextSeq 500 Mid Output kit V2 (Illumina).

The resulting sequences were trimmed and filtered using fastp [43] and assembled using the SPAdes assembler [44]. Multi-locus sequence types (MLST) were assigned according to Dingle et al. [45] and the pubMLST database (https://pubMLST.org/campylobacter) [46]. For core genome MLST (cgMLST) analysis, the chewBBACA pipeline, which performs scheme creation and allele calls on complete and draft genomes, was used [47]. In brief, first whole-genome MLST (wgMLST) schemes were created for *C. jejuni* and *C. coli*, using the genomes: NC_002163.1, NC_022351.2, NC_022353.2, NC_009839.1, NC_017279.1, NC_017280.1, NC_018521.1, NC_018709.4, NZ_CP005081.1, and NZ_CP010906.1 as reference for *C. jejuni;* and NZ_CP019977.1, NZ_CP007183.1, NZ_CP035927.1, NZ_CP007179.1, and NZ_CP018900.1 as reference for *C. coli*. Next, alleles were called from each group of reference genomes and paralogous genes were removed from the wgMLST schemes. The core genomes were defined from wgMLST schemes by selecting the loci present in 95% of the genomes. Alleles were called using the core genomes to extract the cgMLST scheme from target genomes. The cgMLST results based on core genome allelic distances were visualized using GrapeTree [48]. 

All raw sequencing data on the *Campylobacter* isolates in this study have been deposited in the Sequence Read Archive (SRA) (https://www.ncbi.nlm.nih.gov/sra) under accession number PRJNA606971. New MLST profiles have been deposited in the pubMLST database (https://pubmlst.org/) with isolate identities: 79179-19182, 79422-79424, 79528, and 79612-79615.

### 4.5. Statistical Analysis

The results from the bacterial cultures were analyzed by Fisher’s Exact test, performed using a statistical program on the Internet website “Social Science Statistics” (https://www.socscistatistics.com). The tests verified the association between on-farm measures and decrease in colonization of *Campylobacter* in chickens. A probability level of *p* < 0.05 was considered statistically significant.

### 4.6. On-Farm Measures

All broiler producers included in this study were visited at the start of the study by one or two veterinarians, and follow-up visits were made to producers A and B. During the visits, the broiler houses were studied, including the design of the buildings, anteroom, hygiene barriers, and the external environment. Possible risk factors were discussed and measures to reduce the proportion of chickens with *Campylobacter* were proposed and implemented. 

Specific measures taken at broiler producer A, where biosecurity was already at a high level, were sock change in addition to the existing shoe change between different compartments, use of separate wheelbarrows for different corridors to transport dead broilers, and cleaning of water pipes in all departments with alternating air and water flushing. At broiler producer B, several additional hygiene barriers were introduced, the farmer made efforts to minimize the number of wild animals around the broiler houses, and the farmyard was scraped and new gravel was laid. In addition, the water pipes were cleaned with alternating air and water flushing and the pH of the drinking water to the chickens was lowered. At broiler producer C, fly screens were installed on the broiler houses after the results of cross-contamination from nearby cattle became known. No physical actions were implemented at producer D, but possible transmission routes and hygiene barriers were discussed with the farmer.

## Figures and Tables

**Table 1:** Number of samples and incidence of *Campylobacter*-positive samples taken from the internal and external environment at broiler producers and results of bacteriological analysis.

Sample Type	No. of Samples	*C. jejuni*	*C. coli*	*C. lari*	*C. lanienae*
*Internal environment*					
Anteroom	19				
Chicken on farm	387	54	3		
Insects (flies)	4				
Ventilation vent	2				
Water	22	2			
*External environment*					
Cattle	16	9		3	
Chicken at slaughter (neck skin)	5	5			
Dog	2	2			
Fallow deer	1				
Farmyard	23	4			
Mouse	5				
Parent flocks	35	7	6	3	
Snail	2				
Transport crates	56	17			
Water	18	8	1		
Wild birds	18			1	
Wild boar	11		4		4
**Total no. of samples**	**626**	**108**	**14**	**7**	**4**

**Table 2:** Distribution of multi-locus sequence type (MLST) profiles and clonal complexes among isolates of *Campylobacter jejuni* (*n* = 235) and *C. coli* (*n* = 21).

Clonal Complex	MLST	No. of Isolates	Broiler Producer	Source Origin
21	**19**	14	A, C, D	Chicken on farm, chicken at slaughter
21	**21**	11	A	Cattle, chicken on farm, chicken at slaughter (caecum and neck skin samples)
22	**22**	3	C	Cattle
42	**42**	3	A	Transport crates
45	**45**	10	A, B	Chicken on farm, chicken at slaughter, water (pond)
45	**137**	2	B	Chicken on farm, chicken at slaughter
21	**148**	9	B	Chicken on farm, parent flocks
257	**257**	58	A, C, D	Cattle, chicken on farm, chicken at slaughter, water (pipes)
283	**267**	8	A	Transport crates
NA	**441**	4	C	Chicken on farm, chicken at slaughter
45	**538**	12	A, B	Chicken on farm, chicken at slaughter
206	**572**	8	A	Chicken on farm, chicken at slaughter, transport crates
45	**583**	16	A, B	Chicken on farm, chicken at slaughter, water (pond)
NA	**693**	1	B	Water (pond)
1332	**696**	2	D	Chicken at slaughter
828	**827**	1	B	Parent flocks
828	**828**	2	B	Parent flocks
828	**829**	2	B	Chicken on farm, chicken at slaughter
48	**918**	16	A	Chicken at slaughter, transport crates
1034	**1033**	10	B, D	Farmyard, chicken at slaughter
NA	**1172**	1	B	Wild boar
45	**1326**	2	A	Cattle
NA	**1397**	4	A	Chicken on farm, chicken at slaughter
828	**1664**	1	B	Wild boar
52	**2066**	2	D	Chicken at slaughter
828	**2178**	5	B	Chicken on farm, chicken at slaughter, parent flocks
NA	**3137**	1	B	Water (pond)
NA	**3923**	3	B	Chicken on farm, chicken at slaughter, parent flocks
952	**4582**	2	B	Chicken at slaughter
828	**4709**	4	B	Chicken on farm, chicken at slaughter
NA	**6309**	1	B	Farmyard
NA	**7435**	5	B	Chicken on farm, dog, parent flocks
1034	**7809**	1	B	Water (pond)
21	**9198**	6	B	Chicken on farm, chicken at slaughter, water (pond)
NA	**9374**	1	B	Farmyard
21	9714 *	10	B	Chicken on farm, chicken at slaughter
1034	9715 *	3	A	Chicken on farm, chicken at slaughter
952	9716 *	1	B	Chicken at slaughter
1034	9717 *	1	B	Chicken at slaughter
NA	9832 *	3	B	Water (pond)
NA	9833 *	1	B	Water (pond)
NA	9834 *	1	B	Chicken at slaughter
48	9868 *	1	A	Chicken at slaughter
NA	9869 *	1	B	Farmyard
NA	9870 *	1	B	Water (pond)
52	9871 *	1	D	Chicken at slaughter
257	9872 *	1	C	Chicken at slaughter

^*^ Novel MLST profile; NA = not assigned; Chicken at slaughter = cecum samples if not stated otherwise.

## References

[1] European Food Safety Authority (EFSA) (2019). Scientific report on the European Union One Health 2018 Zoonoses Report. EFSA J..

[2] Geissler A.L., Bustos Carrillo F., Swanson K., Patrick M.E., Fullerton K.E., Bennett C., Barrett K., Mahon B.E. (2017). Increasing *Campylobacter* Infections, Outbreaks, and Antimicrobial Resistance in the United States, 2004–2012. Clin. Infect. Dis..

[3] Public Health Agency of Sweden (2019). Tabellsamling över Anmälningspliktiga Smittsamma Sjukdomar I Sverige 2018.

[4] Wagenaar J.A., French N.P., Havelaar A.H. (2013). Preventing *Campylobacter* at the source: Why is it so difficult?. Clin. Infect. Dis..

[5] Sundström K. (2015). Samhällskostnader för fem livsmedelsburna sjukdomar i Sverige. AgriFood Policy Brief..

[6] Zambrano L.D., Levy K., Menezes N.P., Freeman M.C. (2014). Human diarrhea infections associated with domestic animal husbandry: A systematic review and meta-analysis. Trans. R. Soc. Trop. Med. Hyg..

[7] Black R.E., Levine M.M., Clements M.L., Hughes T.P., Blaser M.J. (1988). Experimental *Campylobacter jejuni* infection in humans. J. Infect. Dis..

[8] Robinson D.A. (1981). Infective dose of *Campylobacter jejuni* in milk. Br. Med. J. (Clin. Res. Ed.).

[9] Kaakoush N.O., Castano-Rodriguez N., Mitchell H.M., Man S.M. (2015). Global Epidemiology of *Campylobacter* Infection. Clin. Microbiol. Rev..

[10] Hansson I., Pudas N., Harbom B., Engvall E.O. (2010). Within-flock variations of *Campylobacter* loads in caeca and on carcasses from broilers. Int. J. Food Microbiol..

[11] Rosenquist H., Sommer H.M., Nielsen N.L., Christensen B.B. (2006). The effect of slaughter operations on the contamination of chicken carcasses with thermotolerant Campylobacter. Int. J. Food Microbiol..

[12] Stern N.J., Robach M.C. (2003). Enumeration of *Campylobacter* spp. in broiler feces and in corresponding processed carcasses. J. Food Prot..

[13] Hansson I., Gustafsson P., Lahti E., Olsson Engvall E. 25 Years of the Swedish Campylobacter monitoring program. Proceedings of the 18th International Workshop on Campylobacter, Helicobacter and Related Organisms.

[14] Bouwknegt M., van de Giessen A.W., Dam-Deisz W.D., Havelaar A.H., Nagelkerke N.J., Henken A.M. (2004). Risk factors for the presence of *Campylobacter* spp. in Dutch broiler flocks. Prev. Vet. Med..

[15] Ellis-Iversen J., Jorgensen F., Bull S., Powell L., Cook A.J., Humphrey T.J. (2009). Risk factors for *Campylobacter* colonisation during rearing of broiler flocks in Great Britain. Prev. Vet. Med..

[16] Hansson I., Forshell L.P., Gustafsson P., Boqvist S., Lindblad J., Engvall E.O., Andersson Y., Vågsholm I. (2007). Summary of the Swedish *Campylobacter* program in broilers, 2001 through 2005. J. Food Prot..

[17] Hansson I., Sandberg M., Habib I., Lowman R., Engvall E.O. (2018). Knowledge gaps in control of *Campylobacter* for prevention of campylobacteriosis. Transbound Emerg. Dis..

[18] Hansson I., Engvall E.O., Vågsholm I., Nyman A. (2010). Risk factors associated with the presence of *Campylobacter*-positive broiler flocks in Sweden. Prev. Vet. Med..

[19] Mughini Gras L., Smid J.H., Wagenaar J.A., de Boer A.G., Havelaar A.H., Friesema I.H., French N.P., Busani L., van Pelt W. (2012). Risk factors for campylobacteriosis of chicken, ruminant, and environmental origin: A combined case-control and source attribution analysis. PLoS ONE.

[20] Boysen L., Rosenquist H., Larsson J.T., Nielsen E.M., Sorensen G., Nordentoft S., Hald T. (2014). Source attribution of human campylobacteriosis in Denmark. Epidemiol. Infect..

[21] Thépault A., Poezevara T., Quesne S., Rose V., Chemaly M., Rivoal K. (2018). Prevalence of Thermophilic *Campylobacter* in Cattle Production at Slaughterhouse Level in France and Link Between *C. jejuni* Bovine Strains and Campylobacteriosis. Front. Microbiol..

[22] Sondergaard M.S., Josefsen M.H., Lofstrom C., Christensen L.S., Wieczorek K., Osek J., Hoorfar J. (2014). Low-cost monitoring of *Campylobacter* in poultry houses by air sampling and quantitative PCR. J. Food Prot..

[23] Bahrndorff S., Rangstrup-Christensen L., Nordentoft S., Hald B. (2013). Foodborne disease prevention and broiler chickens with reduced *Campylobacter* infection. Emerg. Infect. Dis..

[24] Allen V.M., Burton C.H., Wilkinson D.J., Whyte R.T., Harris J.A., Howell M., Tinker D.B. (2008). Evaluation of the performance of different cleaning treatments in reducing microbial contamination of poultry transport crates. Br. Poult. Sci..

[25] Atterbury R.J., Gigante A.M., Tinker D., Howell M., Allen V.M. (2020). An improved cleaning system to reduce microbial contamination of poultry transport crates in the United Kingdom. J. Appl. Microbiol..

[26] Callicott K.A., Friethriksdottir V., Reiersen J., Lowman R., Bisaillon J.R., Gunnarsson E., Berndtson E., Hiett K.L., Needleman D.S., Stern N.J. (2006). Lack of evidence for vertical transmission of *Campylobacter* spp. in chickens. Appl. Environ. Microbiol..

[27] Public Health Agency of Sweden *Campylobacter* (Sverige nov 2018-jan 2019). https://www.folkhalsomyndigheten.se/smittskydd-beredskap/utbrott/utbrottsarkiv/campylobacter-sverige-2018-/.

[28] Jacobs-Reitsma W.F. (1995). *Campylobacter* bacteria in breeder flocks. Avian. Dis..

[29] Cox N.A., Richardson L.J., Maurer J.J., Berrang M.E., Fedorka-Cray P.J., Buhr R.J., Byrd J.A., Lee M.D., Hofacre C.L., O’Kane P.M. (2012). Evidence for horizontal and vertical transmission in *Campylobacter* passage from hen to her progeny. J. Food Protect..

[30] Näther G., Alter T., Martin A., Ellerbroek L. (2009). Analysis of risk factors for *Campylobacter* species infection in broiler flocks. Poult. Sci..

[31] The Institute of Environmental Science and Research Ltd (2015). Notifiable Diseases in New Zealand: Annual Report Porirua, New Zealand. https://surv.esr.cri.nz/PDF_surveillance/AnnualRpt/AnnualSurv/2015/2015AnnualReportFinal.pdf.

[32] Nilsson A., Johansson C., Skarp A., Kaden R., Bertilsson S., Rautelin H. (2018). Survival of *Campylobacter jejuni* and *Campylobacter coli* water isolates in lake and well water. APMIS.

[33] Trigui H., Thibodeau A., Fravalo P., Letellier A., Faucher S.P. (2015). Survival in water of *Campylobacter jejuni* strains isolated from the slaughterhouse. Springerplus.

[34] Public Health Agency of Sweden (2017). Epidemiologisk Typning av Campylobacterisolat Insamlade Vecka 11 2017 (Epidemiological Typing of Campylobacter Isolates Collected Week 11 during 2017).

[35] Public Health Agency of Sweden (2017). Epidemiologisk Typning av Campylobacterisolat Insamlade Vecka 34 2017 (Epidemiological Typing of Campylobacter Isolates Collected Week 34 during 2017).

[36] Dryselius R., Jernberg C., Swedish Food Agency & Public Health Agency of Sweden (2019). Campylobacter från Butik och Klinik (Campylobacter from Store and Clinic).

[37] Joensen K.G., Kiil K., Gantzhorn M.R., Nauerby B., Engberg J., Holt H.M., Nielsen H.L., Petersen A.M., Kuhn K.G., Sando G. (2020). Whole-Genome Sequencing to Detect Numerous *Campylobacter jejuni* Outbreaks and Match Patient Isolates to Sources, Denmark, 2015–2017. Emerg. Infect. Dis..

[38] Public Health Agency of Sweden (2018). Epidemiologisk Typning Av Campylobacterisolat Insamlade Vecka 11 2018 ((Epidemiological Typing of Campylobacter Isolates Collected Week 11 during 2018).

[39] Battersby T., Walsh D., Whyte P., Bolton D. (2017). Evaluating and improving terminal hygiene practices on broiler farms to prevent *Campylobacter* cross-contamination between flocks. Food Microbiol..

[40] Ferrari S., Frosth S., Svensson L., Fernström L.L., Skarin H., Hansson I. (2019). Detection of *Campylobacter* spp. in water by dead-end ultrafiltration and application at farm level. J. Appl. Microbiol..

[41] The Swedish Poultry Meat Association All-time low prevalence of *Campylobacter*. https://svenskfagel.se/all-time-low-prevalence-of-campylobacter/.

[42] Public Health Agency of Sweden Campylobacterinfektion. https://www.folkhalsomyndigheten.se/folkhalsorapportering-statistik/statistik-a-o/sjukdomsstatistik/campylobacterinfektion/?t=county.

[43] Chen S., Zhou Y., Chen Y., Gu J. (2018). fastp: An ultra-fast all-in-one FASTQ preprocessor. Bioinformatics.

[44] Bankevich A., Nurk S., Antipov D., Gurevich A.A., Dvorkin M., Kulikov A.S., Lesin V.M., Nikolenko S.I., Pham S., Prjibelski A.D. (2012). SPAdes: A new genome assembly algorithm and its applications to single-cell sequencing. J. Comput. Biol..

[45] Dingle K.E., Colles F.M., Wareing D.R., Ure R., Fox A.J., Bolton F.E., Bootsma H.J., Willems R.J., Urwin R., Maiden M.C. (2001). Multilocus sequence typing system for *Campylobacter jejuni*. J. Clin. Microbiol..

[46] Jolley K.A., Bray J.E., Maiden M.C.J. (2018). Open-access bacterial population genomics: BIGSdb software, the PubMLST.org website and their applications. Wellcome Open Res..

[47] Silva M., Machado M.P., Silva D.N., Rossi M., Moran-Gilad J., Santos S., Ramirez M., Carrico J.A. (2018). chewBBACA: A complete suite for gene-by-gene schema creation and strain identification. Microb. Genom..

[48] Zhou Z., Alikhan N.F., Sergeant M.J., Luhmann N., Vaz C., Francisco A.P., Carrico J.A., Achtman M. (2018). GrapeTree: Visualization of core genomic relationships among 100,000 bacterial pathogens. Genome Res..

